# Exploring the relationship between per- and polyfluoroalkyl substances exposure and rheumatoid arthritis risk using interpretable machine learning

**DOI:** 10.3389/fpubh.2025.1581717

**Published:** 2025-06-03

**Authors:** Zhi Li, Xinping Xu, Ke Zhang

**Affiliations:** ^1^Nanjing Jiangbei Hospital, Affiliated Nanjing Jiangbei Hospital of Xinglin College, Nantong University, Nanjing, Jiangsu, China; ^2^Huai'an No. 3 People's Hospital, Huaian Second Clinical College of Xuzhou Medical University, Huaian, Jiangsu, China; ^3^Huai'an TCM Hospital Affiliated to Nanjing University of Chinese Medicine, Huaian, Jiangsu, China

**Keywords:** machine learning, rheumatoid arthritis, PFAS, SHAP, environmental pollution

## Abstract

**Background:**

Rheumatoid arthritis is a chronic autoimmune disease influenced by environmental exposures, including per- and polyfluoroalkyl substances (PFAS). Although previous studies have suggested links between PFAS and rheumatoid arthritis risk, none have used interpretable machine learning models for prediction. This study aimed to develop such a model to assess risk based on PFAS exposure.

**Methods:**

We analyzed data from 11,705 participants in the National Health and Nutrition Examination Survey (2003–2018). Twelve machine learning algorithms were evaluated using metrics including area under the curve (AUC), accuracy, sensitivity, specificity, and F_1_ score. Key predictors were identified using SHapley Additive exPlanations (SHAP). Partial dependence plots and locally weighted scatterplot smoothing (LOWESS) curves were used to examine non-linear associations and exposure thresholds. A web-based risk calculator was developed to enhance clinical and public health applicability.

**Results:**

CatBoost showed the best performance (AUC: 0.82; Accuracy: 74%; F_1_ score: 0.62) and was selected for further interpretation. SHAP analysis identified perfluorooctane sulfonic acid (PFOS) and 2-(N-Methyl-perfluorooctane sulfonamido) acetic acid (MPAH) as major contributors to risk prediction. PFOS exhibited a U-shaped relationship with increased risk above 15.10 ng/ml, while MPAH showed a risk transition at 0.22 ng/ml. Waterfall plots illustrated the contribution of individual exposures. The interactive web-based calculator allows users to input PFAS levels and receive personalized rheumatoid arthritis risk estimates. It is freely available on Hugging Face Spaces (https://huggingface.co/spaces/Machine199710/RA_ML).

**Conclusions:**

This study demonstrates the potential of machine learning to predict rheumatoid arthritis risk based on PFAS exposure. The identified non-linear patterns provide insights into environmental contributions to disease risk and may inform future prevention strategies.

## Introduction

Rheumatoid arthritis (RA) is a chronic autoimmune disease with variable global prevalence, more common in developed and urban regions, and disproportionately affecting women at a ratio of approximately 3:1 (1, 2). Despite significant advances in treatment, challenges such as delayed diagnosis, disease heterogeneity, and limited access to care in low-resource settings continue to adversely affect patient outcomes (3). In this context, machine learning (ML) has emerged as a promising approach to address these challenges by leveraging complex datasets from omics, imaging, and clinical records (4–6). Liu et al. (4) used ML to identify six diagnostic genes, achieving high accuracy (AUC: 0.996) for diagnosing atherosclerosis (AS) with RA. ML offers the potential to identify novel biomarkers and improve early diagnostic accuracy (7). It also enables personalized treatment strategies, providing new opportunities to enhance the management of RA (8). However, ensuring clinical applicability requires overcoming challenges such as bias and limited generalizability in current models.

Per- and polyfluoroalkyl substances (PFAS) are synthetic chemicals commonly used in industrial and consumer products due to their stability and resistance to degradation (9, 10). However, these properties have also led to their accumulation in the environment and human body (11), where they are associated with various health risks, including immune dysfunction and metabolic disorders (12, 13). PFAS exposure has also been linked to RA (14). For instance, Qiao et al. (14) reported a negative association between PFAS mixture exposure and RA in females. Despite these findings, most studies have relied on traditional statistical methods, which may not fully capture the complex relationships between PFAS exposure and RA (15). While ML has been used to study other environmental factors like heavy metals in relation to RA (16), it has not yet been applied to explore the complex relationships between PFAS exposure and RA or to identify potential patterns and interactions. Using ML in this context could provide valuable insights and improve the prediction and management of RA risk.

In this study, we utilized PFAS exposure data from the 2003 to 2018 National Health and Nutrition Examination Survey (NHANES) to develop ML models for predicting RA. We compared multiple ML algorithms using metrics such as AUC, accuracy, sensitivity, and F_1_ score to identify the most effective model. To enhance interpretability, we applied SHapley Additive exPlanations (SHAP), and Partial Dependence Analysis (PDA) to identify key PFAS associated with RA risk. Additionally, we investigated the relationships between these key PFAS and RA risk, exploring linear and non-linear patterns, such as U-shaped associations, and determined clinically relevant cutoff values. To facilitate the translation of our research findings into a practical tool, we developed and deployed a user-friendly, web-based calculator using the Gradio library. This calculator allows users to input individual risk factors, including PFAS exposure levels, and obtain a personalized RA risk prediction. The calculator is publicly available on Hugging Face Spaces (https://huggingface.co/spaces/Machine199710/RA_ML), providing an accessible resource for both researchers and the general public.

## Methods

### Study participants

The National Health and Nutrition Examination Survey (NHANES), conducted by the National Center for Health Statistics under the CDC, is a cross-sectional survey assessing the health and nutrition of non-institutionalized U.S. residents. This study utilized data from seven NHANES cycles (2003–2018) comprising 80,312 participants. After excluding 68,607 individuals due to missing RA status, serum PFAS measurements, or incomplete covariate data, a final sample of 11,705 participants was included. Missing covariates with < 20% missingness were imputed using the K-Nearest Neighbors (KNN) algorithm with *k* = 5, implemented via *VIM* R package. PFAS variables had no missing values and were therefore excluded from imputation. For variables that underwent imputation, differences in summary statistics before and after imputation were minimal (e.g., BMI mean: 29.218 before vs. 29.206 after), suggesting negligible impact on the overall data structure. RA status was determined through self-reported diagnoses by healthcare professionals, and the participant selection workflow is shown in Supplementary Figure S1. All participants provided written informed consent, and the study was approved by the National Center for Health Statistics Research Ethics Review Board.

### PFAS concentration

Serum PFAS levels were quantified using online solid-phase extraction coupled with high-performance liquid chromatography-turboionspray ionization-tandem mass spectrometry (online SPE-HPLC-TIS-MS/MS), with detailed methods available on the NHANES website (https://wwwn.cdc.gov/Nchs/Data/Nhanes/Public/2017/DataFiles/PFAS_J.htm). The PFAS analyzed included 2-(N-Methyl-perfluorooctane sulfonamido) acetic acid (MPAH), perfluorodecanoic acid (PFDE), perfluorohexane sulfonic acid (PFHxS), perfluorononanoic acid (PFNA), perfluorooctane sulfonic acid (PFOS), perfluorooctanoic acid (PFOA), and perfluoroundecanoic acid (PFUA). For data collected between 2013 and 2018, total PFOS and PFOA concentrations were calculated by summing the concentrations of their respective isomers: linear (n-PFOA) and branched (Sb-PFOA) for PFOA, and linear (n-PFOS) and monomethyl branched (Sm-PFOS) for PFOS. Pearson correlation coefficients among the seven PFAS were calculated and visualized in a heatmap to illustrate their positive and negative correlations.

### Covariates

The covariates included in this study were age, gender, race, education level, Poverty-to-Income Ratio (PIR), body mass index (BMI), smoking status, and alcohol consumption. Race was categorized as Mexican American, other Hispanic, non-Hispanic White, non-Hispanic Black, and other. Education level was grouped into two categories: high school or lower, and more than high school. PIR, representing income as an independent variable, was calculated by dividing household income by the poverty threshold for the specific NHANES survey year. Alcohol consumption was defined as having consumed at least 12 alcoholic drinks in one's lifetime, where one drink was equivalent to a 12 oz. beer, a 5 oz. glass of wine, or 1.5 oz. of liquor. Smoking status was defined as having smoked at least 100 cigarettes during one's lifetime. To ensure the reliability of the model, variance inflation factors (VIFs) were used to assess multicollinearity among covariates, with values below 10 indicating no significant multicollinearity.

### Data preprocessing and construction of ML models

**Figure 1:**
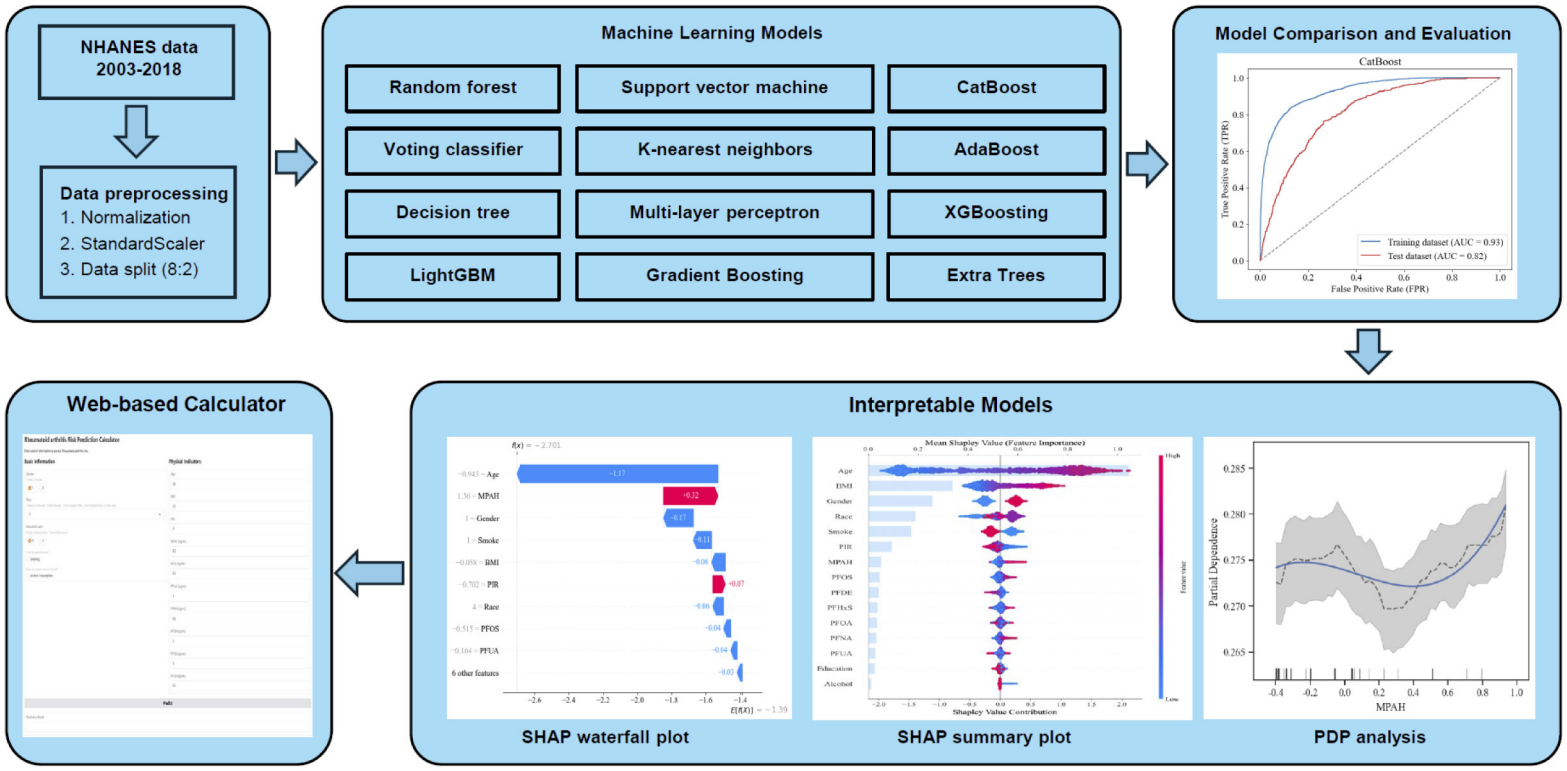
Workflow of the ML analysis for predicting RA risk based on PFAS exposure. The figure shows the workflow for analyzing PFAS exposure and RA risk using ML models. NHANES data (2003–2018) underwent preprocessing, and 12 ML algorithms were evaluated. CatBoost achieved the best performance (AUC 0.82). Feature importance was analyzed using SHAP analysis, while PDA and SHAP visualizations explored non-linear relationships and individual predictions.

This study initially included 15 variables, comprising 10 continuous and five categorical features for ML analysis. Continuous variables were standardized using the “StandardScaler” to ensure consistency across the dataset. The pre-processed data was then split into training (80%, *n* = 9,364) and testing (20%, *n* = 2,341) subsets. Hyperparameter tuning and internal validation were performed using 10-fold cross-validation within the training set. To explore the relationship between PFAS exposure and RA, 12 ML models were tested: AdaBoost (AB), CatBoost (CB), Decision Tree (DT), Extra Trees (ET), K-Nearest Neighbors (KNN), Gradient Boosting (GB), LightGBM (LGB), Multi-Layer Perceptron (MLP), Random Forest (RF), Support Vector Machine (SVM), Voting Classifier (VC), and XGBoost (XGB). Grid search with 10-fold cross-validation was employed to optimize model hyperparameters, and the best configurations (Supplementary Table S1) were applied to the final models. Figure 1 outlines the machine learning workflow used in this study, while model performance was evaluated to identify the most effective approach for predicting RA based on PFAS exposure.

### Evaluation of ML models

To assess the performance of each machine learning model, a variety of metrics were used, including the receiver operating characteristic (ROC) curve, area under the curve (AUC), accuracy, sensitivity (recall), specificity, false-positive rate (FPR), false-negative rate (FNR), positive predictive value (PPV), negative predictive value (NPV), and F_1_ score.

### Interpretation of ML models

Partial Dependence Analysis (PDA) was conducted to investigate how changes in key PFAS affect the model's predictions while keeping other variables constant. Using the “*partial_dependence*” function from “*scikit-learn*” and spline interpolation, the analysis generated smoothed curves to represent the relationship between PFAS levels and RA risk. Additionally, a rug plot was included to display the data distribution, providing further insight into how the model responds to varying feature values.

SHapley Additive exPlanations (SHAP) were applied using the “*TreeExplainer*” for the “*cb_model*” to analyze feature contributions in the testing dataset. A summary plot displayed features ranked by importance and SHAP values, while a decision plot visualized their overall impact on predictions. Additionally, a waterfall plot illustrated individual feature contributions for a specific prediction. SHAP values were also plotted using a scatter plot with a locally weighted scatter plot smoothing (LOWESS) curve to identify trends, marking critical thresholds where features transitioned between positive and negative contributions.

### Web-based calculator development

To translate our findings into a practical tool, we developed a web-based calculator using the Gradio library in Python. Gradio allows for the rapid creation of interactive web interfaces for machine learning models. The calculator's user interface includes input fields for the key predictors identified in our analysis: age, gender, race, education level, smoking status, alcohol consumption, BMI, and serum levels of MPAH, PFDE, PFHxS, PFNA, PFOA, PFOS, and PFUA. The interface utilizes radio buttons for gender and education level, a dropdown menu for race, checkboxes for smoking and alcohol status, and numerical input fields for the remaining variables. Upon clicking a “Predict” button, the user-provided inputs are preprocessed (including standardization of numerical features using the same StandardScaler used during model training), fed into the trained CatBoost model, and the predicted RA risk (probability and risk level) is displayed. The calculator provides an intuitive and accessible way for users to assess their potential RA risk based on their individual characteristics and PFAS exposure levels. The source code and deployment details are available on Hugging Face Spaces (https://huggingface.co/spaces/Machine199710/RA_ML).

### Statistical analysis

For continuous variables, results were expressed as mean and standard deviation (SD), while categorical variables were presented as counts and percentages. Group comparisons for demographic features and PFAS concentrations between RA and non-RA participants were conducted using *t*-tests for continuous data and Chi-square tests for categorical data. Statistical analyses were performed with Python (version 3.9.19) and R (version 4.4.0), with significance defined at *p* < 0.05.

## Results

### Characteristics of study participants

Among the 11,705 participants, as shown in Table 1, the mean age was 50.1 years, with 49.2% male and 50.8% female. The racial distribution included 16.2% Mexican American, 8.8% other Hispanic, 45.1% non-Hispanic White, 20.7% non-Hispanic Black, and 9.2% other. Education levels were nearly evenly split, with 48.1% having a high school education or lower. Mean BMI was 29.2 kg/m^2^, and 54.4% had a history of smoking, while 94.0% reported alcohol consumption. RA participants were older (62.3 vs. 45.4 years, *p* < 0.001), more likely female (59.2% vs. 47.6%, *p* < 0.001), and predominantly non-Hispanic White (54.4% vs. 41.6%, *p* < 0.001) compared to non-RA participants. RA participants also had higher BMI (30.9 vs. 28.5 kg/m^2^, *p* < 0.001) and elevated levels of several PFAS, including MPAH, PFHxS, PFNA, PFOA, and PFOS (all *p* < 0.005). However, no significant differences were observed for PFDE or PFUA levels.

**Table 1:** Baseline characteristics of participants.

**Characteristics**	**Non-RA (*N* = 8,458)**	**RA (*N* = 3,247)**	**Total (*N* = 11,705)**	***p*-Value**
Age, year	45.4 (17.3)	62.3 (13.9)	50.1 (18.1)	< 0.001
**Gender**, ***n*** **(%)**				
Male	4,432 (52.4%)	1,325 (40.8%)	5,757 (49.2%)	< 0.001
Female	4,026 (47.6%)	1,922 (59.2%)	5,948 (50.8%)	
**Race**, ***n*** **(%)**				
Mexican American	1,532 (18.1%)	362 (11.1%)	1,894 (16.2%)	< 0.001
Other Hispanic	769 (9.1%)	262 (8.1%)	1,031 (8.8%)	
Non-Hispanic White	3,517 (41.6%)	1,766 (54.4%)	5,283 (45.1%)	
Non-Hispanic Black	1,764 (20.9%)	659 (20.3%)	2,423 (20.7%)	
Other	876 (10.4%)	198 (6.1%)	1,074 (9.2%)	
**Education**, ***n*** **(%)**				
High school or lower	3,901 (46.1%)	1,724 (53.1%)	5,625 (48.1%)	< 0.001
More than high school	4,557 (53.9%)	1,523 (46.9%)	6,080 (51.9%)	
Weight, kg	80.9 (21.4)	84.8 (22.7)	82.0 (21.8)	< 0.001
Height, cm	168 (10.0)	165 (10.2)	167 (10.1)	< 0.001
BMI, kg/m^2^	28.5 (6.63)	30.9 (7.42)	29.2 (6.94)	< 0.001
Smoke, *n* (%)	4,862 (57.5%)	1,509 (46.5%)	6,371 (54.4%)	< 0.001
Alcohol, *n* (%)	8,010 (94.7%)	2,990 (92.1%)	11,000 (94.0%)	< 0.001
MPAH, ng/ml	0.317 (0.478)	0.399 (1.09)	0.340 (0.706)	< 0.001
PFDE, ng/ml	0.366 (0.850)	0.354 (0.468)	0.363 (0.763)	0.87
PFHxS, ng/ml	2.19 (2.83)	2.29 (2.56)	2.22 (2.76)	< 0.001
PFNA, ng/ml	1.14 (1.09)	1.24 (1.30)	1.17 (1.16)	0.005
PFOA, ng/ml	3.24 (3.05)	3.46 (3.21)	3.30 (3.10)	< 0.001
PFOS, ng/ml	13.6 (21.5)	15.9 (20.4)	14.2 (21.3)	< 0.001
PFUA, ng/ml	0.262 (1.10)	0.242 (0.376)	0.256 (0.952)	0.701

### Trends and correlations of PFAS concentrations (2003–2018)

As shown in Table 2, serum PFAS concentrations generally declined across NHANES cycles from 2003 to 2018. Notable reductions included PFOS, which dropped from 25.7 to 7.18 ng/ml, and PFOA, which decreased from 4.42 to 1.77 ng/ml. Similar trends were observed for MPAH, PFDE, PFHxS, PFNA, and PFUA, likely reflecting regulatory restrictions and reduced industrial use of PFAS. Pearson correlation analysis (Supplementary Figure S2) revealed a strong correlation between PFDE and PFUA (*r* = 0.77) and moderate correlations of PFUA with PFOS (*r* = 0.47) and PFOA with PFNA (*r* = 0.47). In contrast, MPAH exhibited weak correlations with most other PFAS (*r* < 0.2), indicating distinct exposure pathways or unique environmental behaviors for certain compounds.

**Table 2:** Serum concentration of PFAS of eight NHANES cycles.

**PFAS**	**Cycles of NHANES**									***p*-Value**
	**2003–2005 (*****N*** = **1,339)**	**2005–2006 (*****N*** = **1,368)**	**2007–2008 (*****N*** = **1,632)**	**2009–2010 (*****N*** = **1,656)**	**2011–2012 (*****N*** = **1,383)**	**2013–2014 (*****N*** = **1,469)**	**2015–2016 (*****N*** = **1,503)**	**2017–2018 (*****N*** = **1,355)**	**Total (*****N*** = **11,705)**	
MPAH, ng/ml	0.557 (1.52)	0.597 (0.737)	0.466 (0.657)	0.318 (0.506)	0.234 (0.371)	0.193 (0.310)	0.172 (0.285)	0.195 (0.318)	0.340 (0.706)	< 0.001
PFDE, ng/ml	0.337 (0.340)	0.533 (0.779)	0.396 (0.720)	0.397 (0.696)	0.333 (0.635)	0.346 (1.44)	0.275 (0.481)	0.282 (0.339)	0.363 (0.763)	< 0.001
PFHxS, ng/ml	2.62 (2.75)	2.41 (2.74)	2.97 (3.88)	2.25 (2.72)	1.89 (2.62)	2.05 (2.24)	1.75 (1.88)	1.70 (2.39)	2.22 (2.76)	< 0.001
PFNA, ng/ml	1.23 (1.21)	1.42 (1.34)	1.51 (1.36)	1.56 (1.49)	1.20 (0.972)	0.902 (0.707)	0.824 (0.754)	0.601 (0.549)	1.17 (1.16)	< 0.001
PFOA, ng/ml	4.42 (3.66)	4.66 (3.60)	4.83 (3.78)	3.53 (2.30)	2.58 (1.93)	2.50 (3.38)	2.00 (1.72)	1.77 (1.38)	3.30 (3.10)	< 0.001
PFOS, ng/ml	25.7 (25.6)	22.5 (19.0)	18.7 (18.0)	13.2 (15.7)	10.3 (12.4)	9.00 (37.5)	7.93 (8.91)	7.18 (8.10)	14.2 (21.3)	< 0.001
PFUA, ng/ml	0.274 (0.387)	0.301 (0.552)	0.269 (0.470)	0.301 (0.840)	0.271 (0.449)	0.268 (2.33)	0.165 (0.267)	0.197 (0.251)	0.256 (0.952)	< 0.001

### Performance evaluation and comparison of ML models

**Figure 2:**
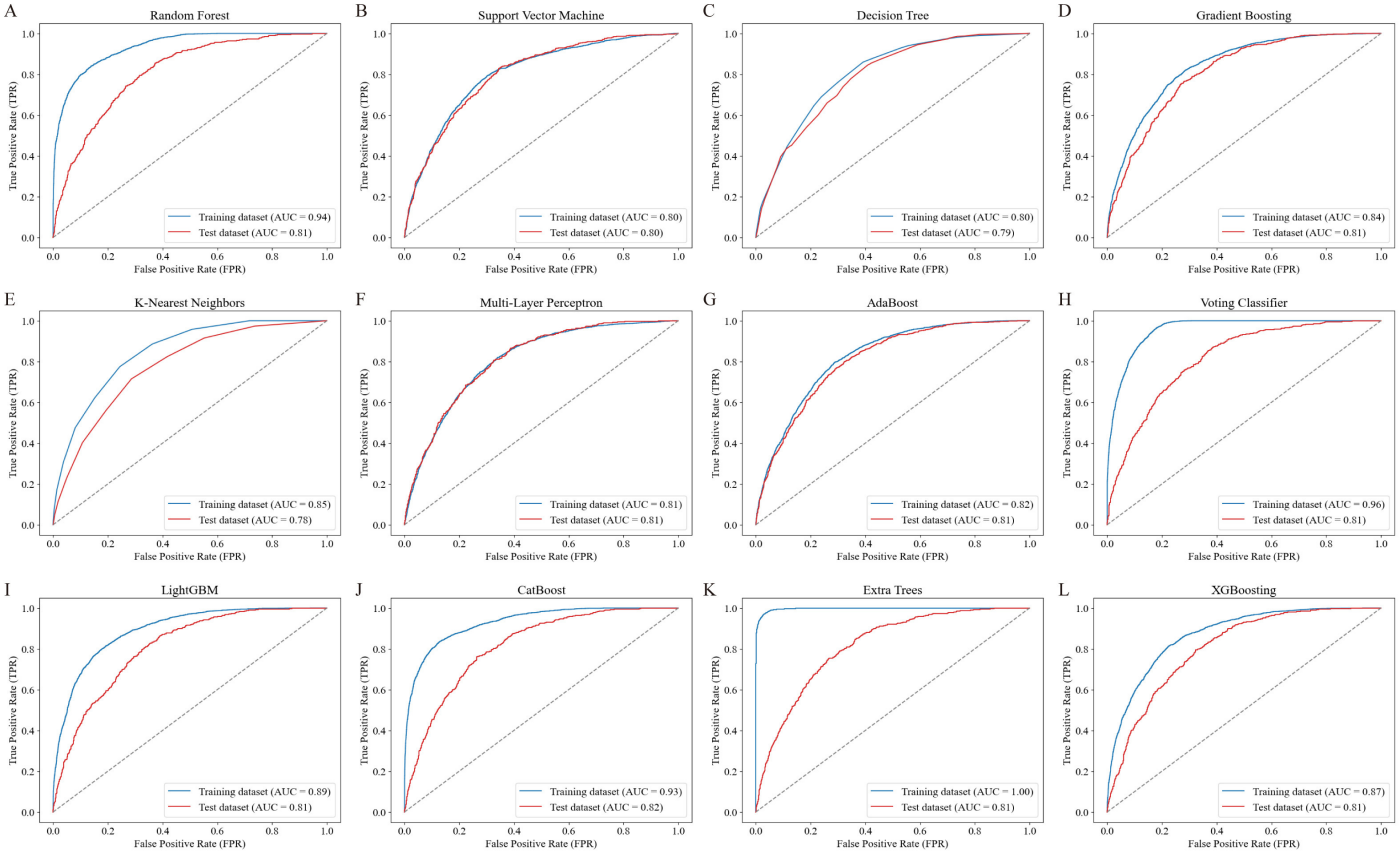
ROC curves of the 12 ML models for predicting RA risk. The ROC curves illustrate the performance of 12 ML models in predicting RA risk based on PFAS exposure. Each panel **(A–L)** represents the training (blue line) and testing (red line) dataset performance for a specific model: **(A)** Random Forest, **(B)** Support Vector Machine, **(C)** Decision Tree, **(D)** Gradient Boosting, **(E)** K-Nearest Neighbors, **(F)** Multi-Layer Perceptron, **(G)** AdaBoost, **(H)** Voting Classifier, **(I)** LightGBM, **(J)** CatBoost, **(K)** Extra Trees, and **(L)** XGBoost. The area under the curve (AUC) values for both training and testing datasets are shown, with CatBoost achieving the highest test dataset AUC of 0.82, demonstrating favorable performance.

Figure 2 presents the ROC curves for 12 ML models, comparing their performance on the training and testing datasets. Among the models, CB achieved the highest test dataset AUC (0.82), demonstrating its strong ability to generalize effectively. Table 3 summarizes the performance metrics for the 12 ML models, including AUC, accuracy, sensitivity (recall), specificity, FPR, FNR, PPV, NPV, and F_1_ score. Among these models, CB achieved the highest testing accuracy (74%) and the highest F_1_ score (0.62), with a strong AUC (0.82). It also maintained a good balance between sensitivity (0.76) and specificity (0.73), while minimizing FPR (0.27) and FNR (0.24). Based on these findings, CB was selected as the optimal model for further analysis due to its robust overall performance across multiple metrics, including AUC, accuracy, and F_1_ score.

**Table 3:** Discrimination characteristics among 12 ML models.

**Metrics**	**RF**	**SVM**	**DT**	**GB**	**KNN**	**MLP**	**AB**	**VC**	**LGB**	**CB**	**ET**	**XGB**
AUC	0.81	0.80	0.79	0.81	0.78	0.81	0.81	0.81	0.81	0.82	0.81	0.81
Accuracy (%)	69	70	66	73	71	71	73	69	68	74	74	71
Sensitivity/recall	0.85	0.82	0.85	0.76	0.71	0.80	0.76	0.85	0.87	0.76	0.75	0.79
Specificity	0.63	0.66	0.58	0.72	0.71	0.68	0.71	0.63	0.61	0.73	0.74	0.68
FPR	0.37	0.34	0.42	0.28	0.29	0.32	0.29	0.37	0.39	0.27	0.26	0.32
FNR	0.15	0.18	0.15	0.24	0.29	0.20	0.24	0.15	0.13	0.24	0.25	0.21
PPV	0.47	0.48	0.44	0.51	0.49	0.49	0.50	0.47	0.46	0.52	0.52	0.48
NPV	0.92	0.91	0.91	0.89	0.87	0.90	0.89	0.92	0.92	0.89	0.88	0.90
F_1_ score	0.60	0.60	0.58	0.61	0.58	0.60	0.61	0.61	0.60	0.62	0.61	0.60

AB, AdaBoost; AUC, area under the receiver operator curve; RF, Random Forest; SVM, Support Vector Machine; DT, Decision Tree; GB, Gradient Boosting; KNN, K-Nearest Neighbors; MLP, Multi-Layer Perceptron; VC, Voting Classifier; LGB, LightGBM; CB, CatBoost; ET, Extra Trees; XGB, XGBoost; FPR, false positive rate; FNR, false negative rate; PPV, positive predictive value; NPV, negative predictive value.

Notably, the ET model also demonstrated highly competitive results, with an AUC of 0.81, accuracy of 74%, and F_1_ score of 0.61—closely matching CB's performance. This suggests that ET may serve as a strong alternative in similar modeling tasks.

### Interpretation of the optimal ML model

**Figure 3:**
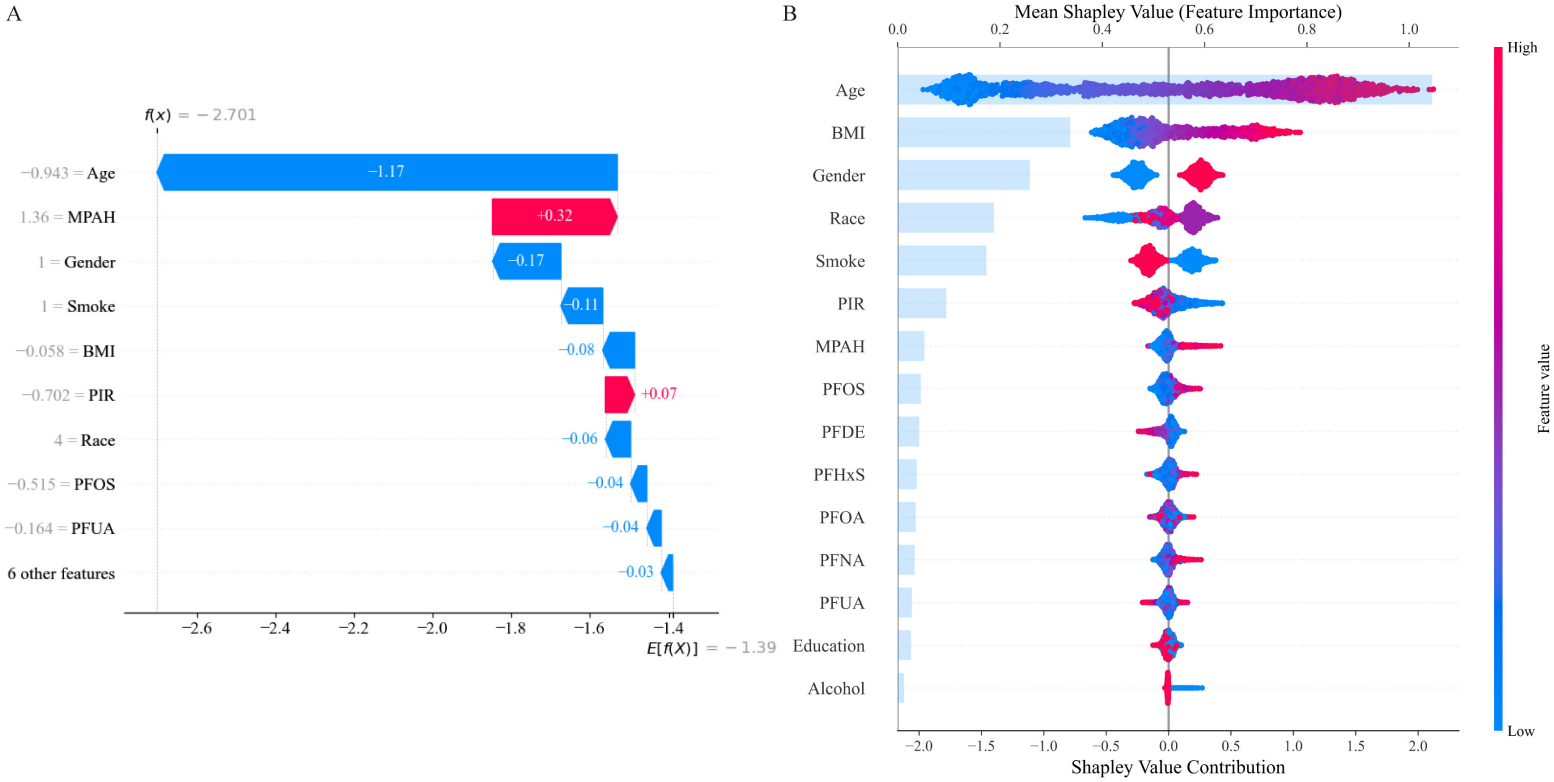
SHAP Analysis for Feature Contributions in the CatBoost Model. **(A)** SHAP waterfall plot illustrates individual feature contributions to RA risk prediction, with age and BMI showing the strongest negative impacts, while MPAH contributes positively. **(B)** SHAP summary plot ranks features by their mean SHAP values across the dataset, highlighting age and BMI as the most influential, with PFOS and MPAH showing moderate contributions depending on their values.

SHAP analysis was employed to visually illustrate how specific features either increased or decreased the risk of RA in the CB model (Figure 3). The waterfall plot (Figure 3A) shows how each feature influenced the model's prediction for this individual, with age and BMI having the strongest negative impacts, while MPAH contributed positively to the risk. The summary plot (Figure 3B) ranks feature by their mean SHAP values across the dataset, with age being the most influential feature overall, followed by BMI, gender, race, and smoking status. Among the PFAS, MPAH had the highest importance with a predominantly positive effect, while PFOS displayed mixed contributions depending on its value. These findings emphasize the combined impact of demographic and PFAS features in both individual and overall model predictions.

**Figure 4:**
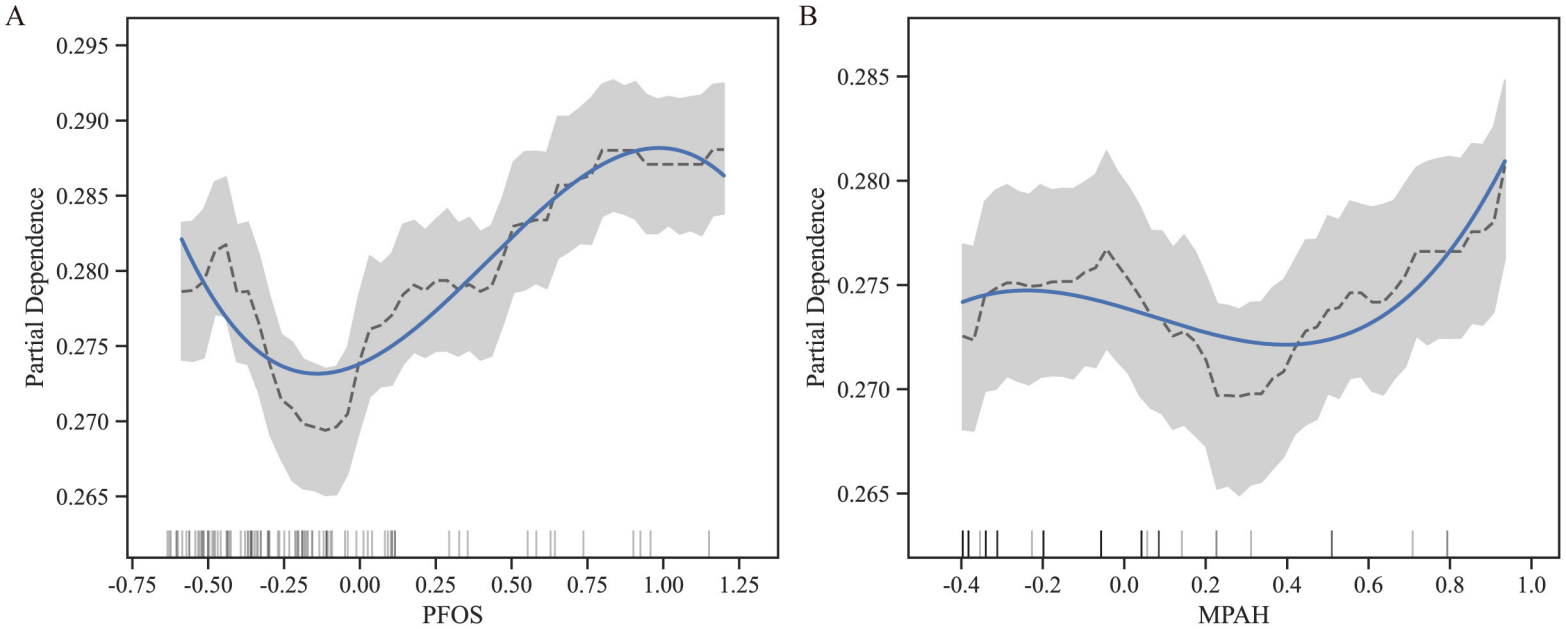
Partial Dependence Plots (PDPs) for PFOS and MPAH. **(A)** PDP for PFOS displays a U-shaped relationship with RA risk, showing decreased risk at moderate levels and increased risk at both low and high concentrations. **(B)** PDP for MPAH illustrates a transition from a slight negative association at lower levels to a positive association at higher levels. The solid blue line represents the fitted relationship, while the dashed black line denotes smoothed trends. The gray shaded area indicates the 95% confidence interval, and the rug plot below each graph illustrates the data distribution along the feature range.

Through SHAP analysis, MPAH and PFOS were identified as the most important PFAS. Subsequently, we conducted further analysis to explore the relationships between these two PFAS and RA risk. Figure 4 presents the Partial Dependence Plots (PDPs) for PFOS (Figure 4A) and MPAH (Figure 4B), illustrating their non-linear effects on RA risk. PFOS exhibits a U-shaped relationship, with moderate levels linked to a lower risk of RA, while both low and high levels are associated with increased risk. MPAH shows a slight negative association with RA risk at lower and moderate concentrations, transitioning to a positive association at higher concentrations.

**Figure 5:**
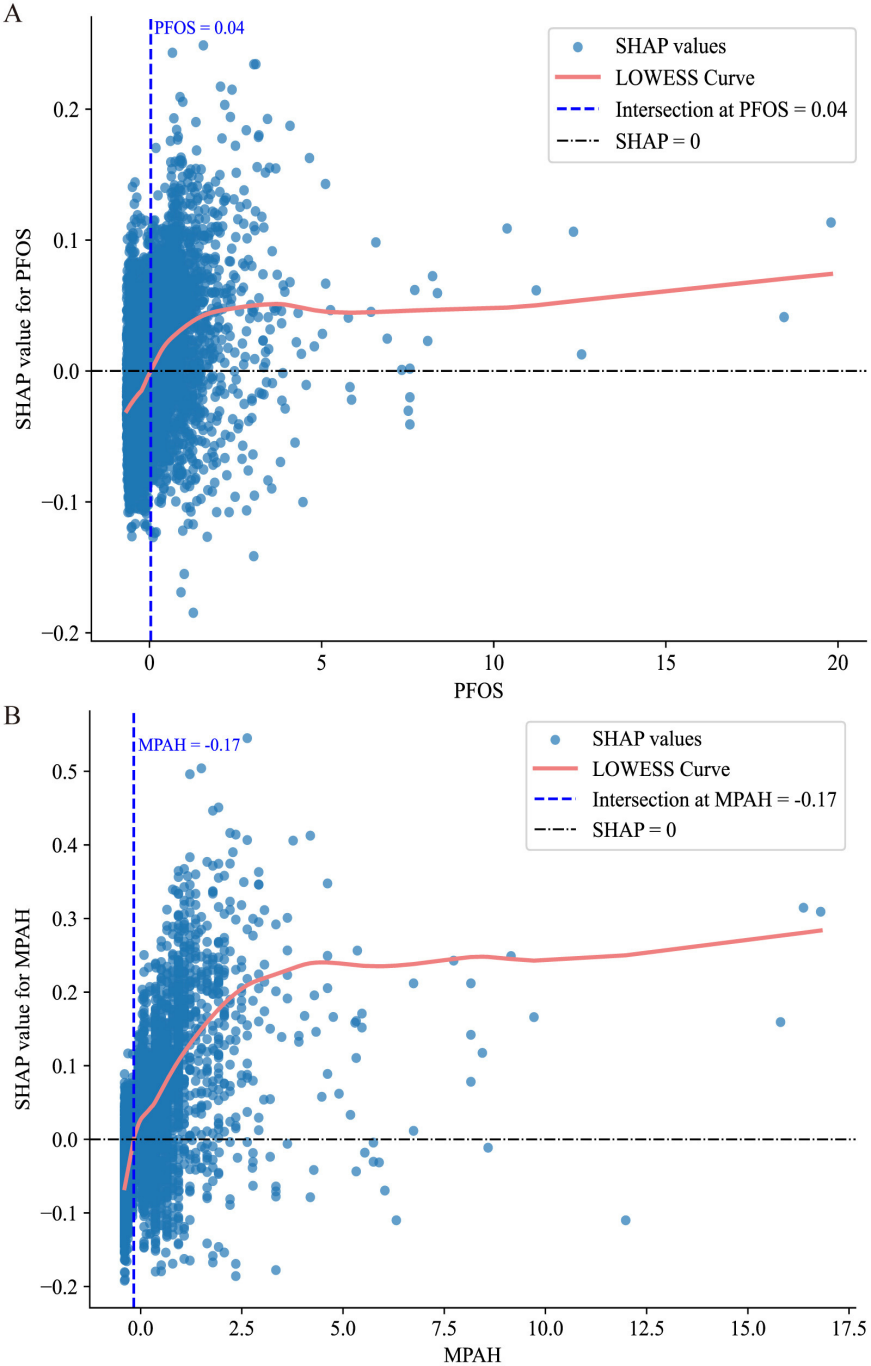
SHAP value scatter plots with LOWESS curves for PFOS and MPAH. **(A)** PFOS: The SHAP values show a transition from negative to positive contributions at a standardized value of 0.04, corresponding to 15.10 ng/ml. **(B)** MPAH: The SHAP values transition from negative to positive contributions at a standardized value of −0.17, corresponding to 0.22 ng/ml. The solid red line represents the LOWESS curve, the dashed blue line indicates the critical threshold, and the black dashed line marks SHAP = 0.

**Figure 6:**
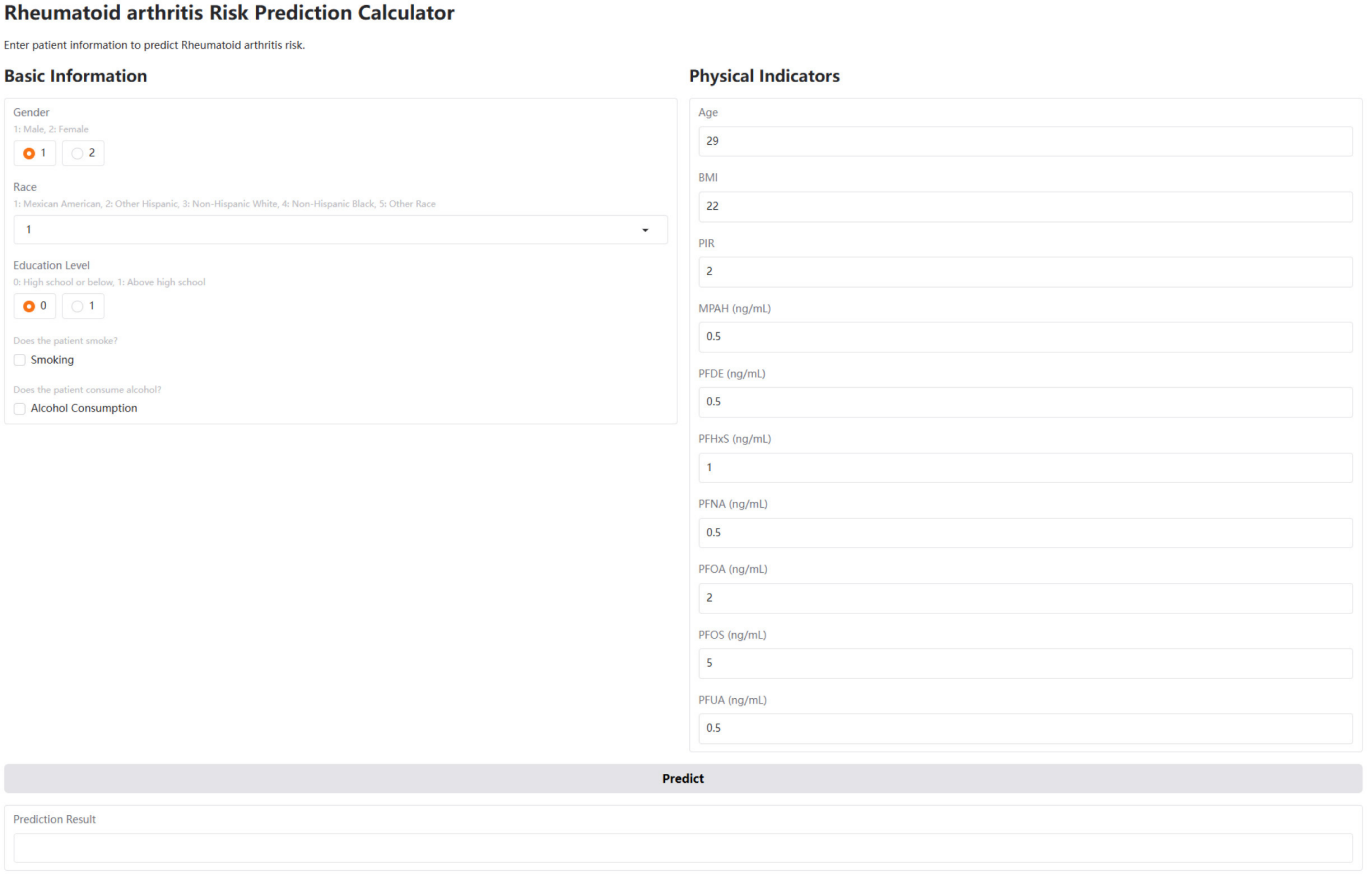
User interface of the interactive web-based calculator for predicting RA risk. The interface is divided into two sections: Basic Information and Physical Indicators. The Basic Information section includes radio buttons for gender (1: male, 2: female) and education level (0: high school or below, 1: above high school), a dropdown menu for race (1: Mexican American, 2: Other Hispanic, 3: Non-Hispanic White, 4: Non-Hispanic Black, 5: Other Race), and checkboxes for smoking and alcohol consumption. The Physical Indicators section includes numerical input fields for age, BMI, PIR, and serum concentrations (in ng/ml) of MPAH, PFDE, PFHxS, PFNA, PFOA, PFOS, and PFUA. A “Predict” button triggers the risk calculation, and the results are displayed in the “Prediction Result” area below.

Figure 5 further supports these findings through SHAP scatter plots with LOWESS curves, showing the transitions from negative to positive contributions. For PFOS, the threshold where the SHAP value shifts occurs at approximately 0.04 (standardized value), corresponding to 15.10 ng/ml in the original scale. For MPAH, the threshold occurs at −0.17 (standardized value), equivalent to 0.22 ng/ml in the original scale. These results underscore the critical roles of PFOS and MPAH in RA risk, revealing complex and non-linear relationships that provide valuable insights for predictive modeling. These findings have been incorporated into an interactive web-based calculator, available at https://huggingface.co/spaces/Machine199710/RA_ML, to facilitate personalized risk assessment (Figure 6).

## Discussion

Using data from the U.S. NHANES (2003–2018), our study introduced an interpretable ML approach to investigate the association between PFAS exposure and RA. This is the first study to apply interpretable ML techniques to examine the link between PFAS exposure and RA risk. Among the 12 models tested, CatBoost demonstrated the best performance, achieving an accuracy of 74%, an F_1_ score of 0.62, and an AUC of 0.82, making it the preferred choice for identifying RA risk. To enhance the interpretability of the model, techniques such as SHAP analysis and Partial Dependence Analysis were employed to assess feature contributions. At the individual level, the SHAP waterfall plot illustrated how specific features influenced predictions, highlighting personalized insights. PFOS and MPAH were identified as the most significant PFAS linked to RA risk, showing non-linear relationships: PFOS exhibited a U-shaped effect with a critical point at 15.10 ng/ml, while MPAH demonstrated a shift from a slight negative to positive association at 0.22 ng/ml. To make our findings readily accessible and usable, we deployed a web-based RA risk calculator. This interactive tool allows users, including clinicians and individuals, to input their own values for the key predictors and obtain an estimated RA risk. This facilitates the practical application of our model and contributes to a more proactive approach to RA risk assessment.

ML has revolutionized medicine by enabling the analysis of large and complex datasets, uncovering patterns and associations that traditional methods often overlook (15, 17). Unlike conventional statistical techniques, ML models excel at capturing non-linear relationships, handling high-dimensional data, and improving predictive accuracy through iterative learning (18). In the context of RA, ML has been employed to address key challenges such as diagnosis, treatment prediction, and biomarker discovery (18–20). Several studies have explored the application of ML in RA. For instance, Liu et al. (4) utilized a combination of RF and least absolute shrinkage and selection operator (LASSO) algorithms to identify immune-related genes for diagnosing AS in RA patients, achieving high diagnostic accuracy (AUC 0.995). Another study focused on treatment response prediction, systematically reviewing ML models used to predict responses to disease-modifying antirheumatic drugs (DMARDs) (21). While promising, many models exhibited unclear or high risks of bias, underscoring the need for improved methodologies and external validation. Additionally, Liu et al. (6) constructed a diagnostic model for RA based on platelet-related genes, demonstrating excellent performance (AUC up to 0.979) and emphasizing the potential of ML in uncovering novel diagnostic markers. Our study also applied 12 ML methods to investigate the relationship between PFAS exposure and RA risk, achieving promising predictive performance. Although the CatBoost model demonstrated relatively strong overall performance, it exhibited a false negative rate of 0.24. In clinical contexts, false negatives are of particular concern, as they may lead to missed diagnoses. Therefore, while the model shows promise, further refinement is needed to reduce the risk of overlooking true RA cases. Beyond optimizing machine learning models, improving their interpretability is equally important, as it facilitates better integration with clinical practice and public health applications.

While CatBoost was selected as the final model due to its overall strong and balanced performance, the Extra Trees (ET) model also demonstrated comparable results. With an AUC of 0.81, accuracy of 74%, and an F_1_ score of 0.61, the ET model performed similarly across key evaluation metrics. These findings highlight the robustness of tree-based ensemble methods in capturing complex, non-linear relationships in high-dimensional environmental health data. Given its competitive performance, ET may serve as a valuable alternative model, especially in scenarios where computational simplicity, model diversity, or interpretability through ensemble averaging is prioritized.

Interpretable ML is critical for bridging the gap between predictive models and practical applications in clinical and public health settings (22–24). By providing transparency, methods like SHAP enable a deeper understanding of how individual features influence model predictions, facilitating trust and actionable insights (25). In RA research, interpretable ML techniques have been used to identify key biomarkers and assess their contributions to disease risk and treatment outcomes. For example, SHAP analysis has been employed to evaluate the impact of clinical features, such as inflammatory markers, on treatment response, enhancing model interpretability (26, 27). In our study, we applied 12 ML algorithms to explore the relationship between PFAS exposure and RA, selecting the CatBoost model for its relatively strong performance. Using SHAP and PDA, we identified PFOS and MPAH as the most influential PFAS and uncovered their non-linear associations with RA risk. For PFOS, the risk increased at levels above 15.10 ng/ml, while MPAH showed a similar threshold effect at 0.22 ng/ml. These findings highlight critical exposure thresholds that warrant further investigation. However, validating these thresholds and understanding the toxicological mechanisms underlying the PFAS-RA relationship require additional experimental studies. The availability of our web-based calculator allows for further exploration of these relationships in a user-friendly format and can help bridge the gap between research findings and practical application.

The non-linear relationships observed between PFOS, MPAH, and RA in our study may reflect the dual immunomodulatory effects of PFAS through intricate molecular and cellular pathways. At lower concentrations, PFAS may interact with immune cells, such as T regulatory (Treg) cells, promoting their activation and function to suppress excessive immune responses (28). This protective mechanism could help maintain immune homeostasis, reducing the risk of inflammation-driven conditions like RA (29). Additionally, PFAS at low levels may modulate signaling pathways, such as the nuclear receptor PPAR-γ (peroxisome proliferator-activated receptor gamma) (30–32), which has anti-inflammatory properties and can inhibit the production of pro-inflammatory cytokines like TNF-α and IL-6 (33).

Conversely, higher concentrations of PFOS and MPAH might overwhelm these regulatory mechanisms, leading to immune dysregulation. PFAS are known to disrupt the NF-κB pathway, a critical regulator of immune responses, driving the overproduction of pro-inflammatory cytokines and chemokines (29, 34). This shift can lead to a chronic inflammatory state, which is a hallmark of RA. Furthermore, PFAS at high levels may interfere with the differentiation and function of immune cells such as macrophages and dendritic cells, skewing them toward a pro-inflammatory phenotype and amplifying the inflammatory cascade (35, 36). Furthermore, PFAS may disrupt mitochondrial function and increase reactive oxygen species (ROS) production, causing oxidative stress. This stress can activate inflammasomes, such as NLRP3, further contributing to the inflammatory milieu (37). Additionally, PFAS may affect lipid metabolism and membrane integrity, potentially altering the signaling processes in immune cells and exacerbating inflammation (38, 39). This U-shaped behavior reflects the balance between protective and harmful effects of PFAS, driven by concentration-dependent impacts on immune pathways and cellular function. Further research is needed to clarify these mechanisms and validate thresholds, offering insights into PFAS contributions to RA and potential interventions.

Additionally, the observed decline in serum PFAS concentrations across NHANES cycles from 2003 to 2018—particularly for compounds such as PFOS and PFOA—likely reflects regulatory actions and changes in industrial practices. While this downward trend is encouraging from a public health standpoint, it introduces analytical complexity when evaluating associations with chronic diseases like RA. Specifically, the temporal mismatch between decreasing PFAS levels and increasing RA prevalence may lead to residual confounding or attenuated associations. These findings underscore the importance of accounting for time as a potential effect modifier or confounder in future research, particularly in studies leveraging cross-sectional datasets like NHANES.

This study has several limitations. First, RA diagnosis was based on self-reported NHANES data, which may introduce recall bias and affect classification accuracy. Second, although NHANES uses a multi-stage stratified sampling design, it may not fully represent the entire U.S. population, potentially limiting generalizability. Third, the absence of external datasets restricts the ability to confirm model performance and reproducibility. While internal validation was performed using 10-fold cross-validation, the lack of external validation using independent datasets limits generalizability. In addition, key RA risk factors such as genetic susceptibility and co-exposure to other environmental pollutants were not included, which may have introduced confounding bias. The temporal mismatch between declining PFAS levels and increasing RA prevalence further complicates interpretation. Finally, since PFAS testing is not routinely conducted in clinical settings, the practical application of the web-based risk calculator may be limited. Future research should address these limitations through clinically confirmed RA diagnoses, incorporation of genetic and environmental covariates, use of external validation cohorts, and consideration of temporal trends and data accessibility.

## Conclusion

In conclusion, this study utilized an interpretable machine learning framework to investigate the association between PFAS exposure and RA using data from the NHANES (2003–2018). Among the 12 tested models, CatBoost demonstrated favorable performance, achieving an AUC of 0.82 and an accuracy of 74%, providing robust predictions and highlighting PFOS and MPAH as key PFAS associated with RA risk. This analysis revealed non-linear relationships for these PFAS, with distinct threshold effects, emphasizing the complexity of their role in RA pathogenesis. These findings underscore the importance of integrating environmental exposure data into predictive models to enhance RA risk assessment and inform public health strategies. Furthermore, the development and deployment of a web-based calculator based on these findings provides a practical tool for individuals and clinicians to assess RA risk, promoting proactive health management.

## Data Availability

The original contributions presented in the study are included in the article/Supplementary material, further inquiries can be directed to the corresponding authors.

## Supplementary material

Supplementary Figure S1 Participant selection flowchart.

Supplementary Figure S2 Correlation heatmap of serum PFAS concentrations.

Supplementary Table S1 Hyperparameters for 12 ML models.

## Glossary

AUC, area under the curve; BMI, body mass index; CB, CatBoost; CDC, Centers for Disease Control and Prevention; DMARDs, disease-modifying antirheumatic drugs; ET, Extra Trees; FNR, false-negative rate; FPR, false-positive rate; GB, Gradient Boosting; KNN, K-Nearest Neighbor; LASSO, least absolute shrinkage and selection operator; LGB, LightGBM; LOWESS, locally weighted scatter plot smoothing; ML, machine learning; MLP, Multi-Layer Perceptron; MPAH, 2-(N-Methyl-perfluorooctane sulfonamido) acetic acid; NF-κB, nuclear factor kappa-light-chain-enhancer of activated B cells; NHANES, national health and nutrition examination survey; NPV, negative predictive value; n-PFOA, linear perfluorooctanoic acid; n-PFOS, linear perfluorooctane sulfonic acid; NLRP3, nucleotide-binding oligomerization domain, leucine-rich repeat and pyrin domain containing 3; PDA, Partial Dependence Analysis; PDP, Partial Dependence Plot; PFAS, Per- and polyfluoroalkyl substances; PFDE, perfluorodecanoic acid; PFHxS, perfluorohexane sulfonic acid; PFNA, perfluorononanoic Acid; PFOA, perfluorooctanoic acid; PFOS, perfluorooctane sulfonic acid; PFUA, perfluoroundecanoic acid; PIR, Poverty-to-Income Ratio; PPAR-γ, peroxisome proliferator-activated receptor gamma; PPV, positive predictive value; RA, rheumatoid arthritis; ROC, receiver operating characteristic; ROS, reactive oxygen species; Sb-PFOA, branched perfluorooctanoic acid; SHAP, SHapley Additive exPlanations; Sm-PFOS, monomethyl branched perfluorooctane sulfonic acid; SPE-HPLC-TIS-MS/MS, solid-phase extraction high-performance liquid chromatography turboionspray ionization tandem mass spectrometry; SVM, Support Vector Machine; Treg, T Regulatory Cells; VC, Voting Classifier; VIF, variance inflation factor; XGB, XGBoost.
