# Supplementary figures and images for: Exploring the relationship between per- and polyfluoroalkyl substances exposure and rheumatoid arthritis risk using interpretable machine learning
Source: Front Public Health. 2025 Jun 3;13:1581717. doi: 10.3389/fpubh.2025.1581717 (PMC12170615; doi:10.3389/fpubh.2025.1581717)

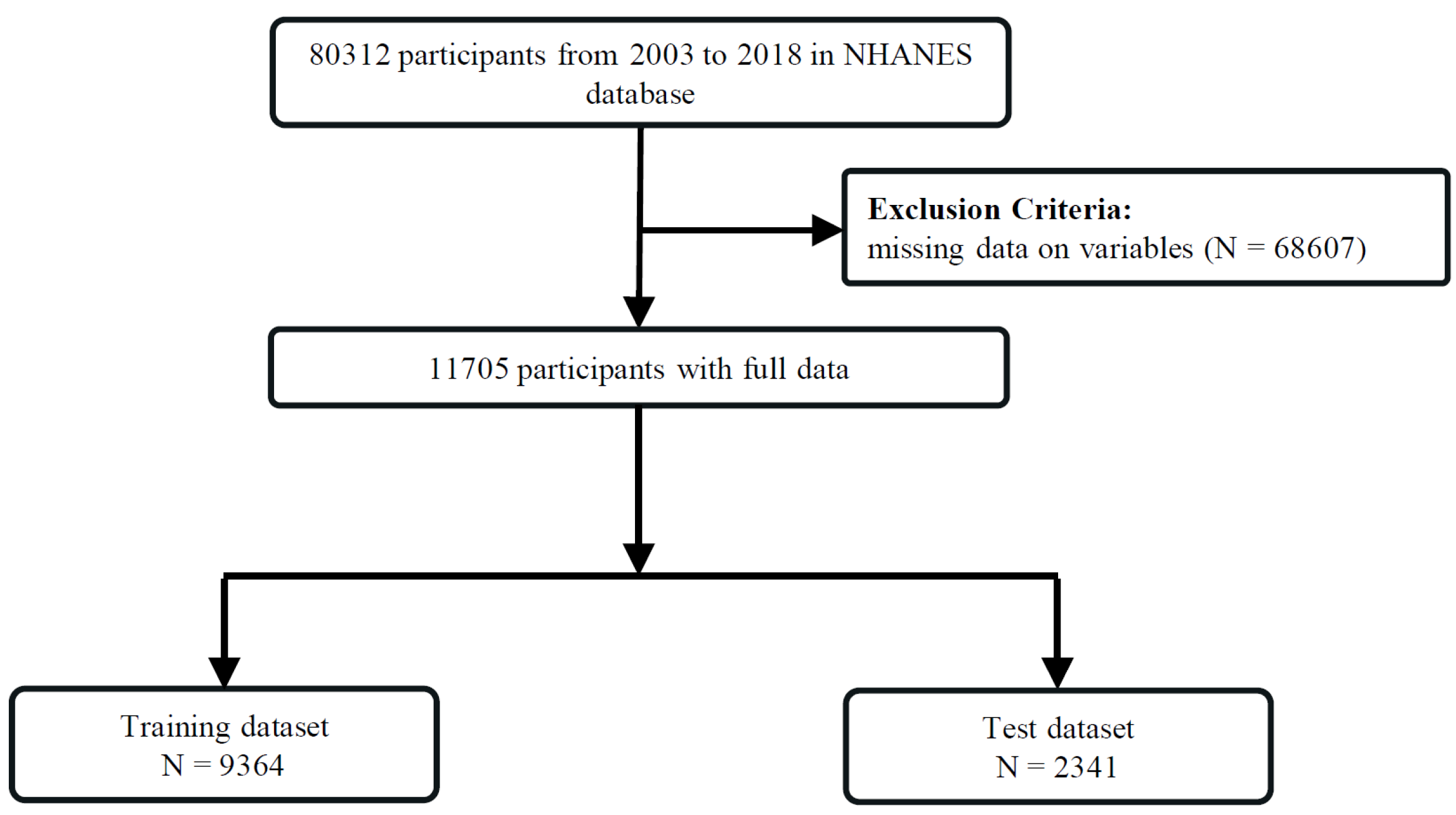

Supplement: Supplementary Figure S1 — Participant selection flowchart. [file Image_1.tif]

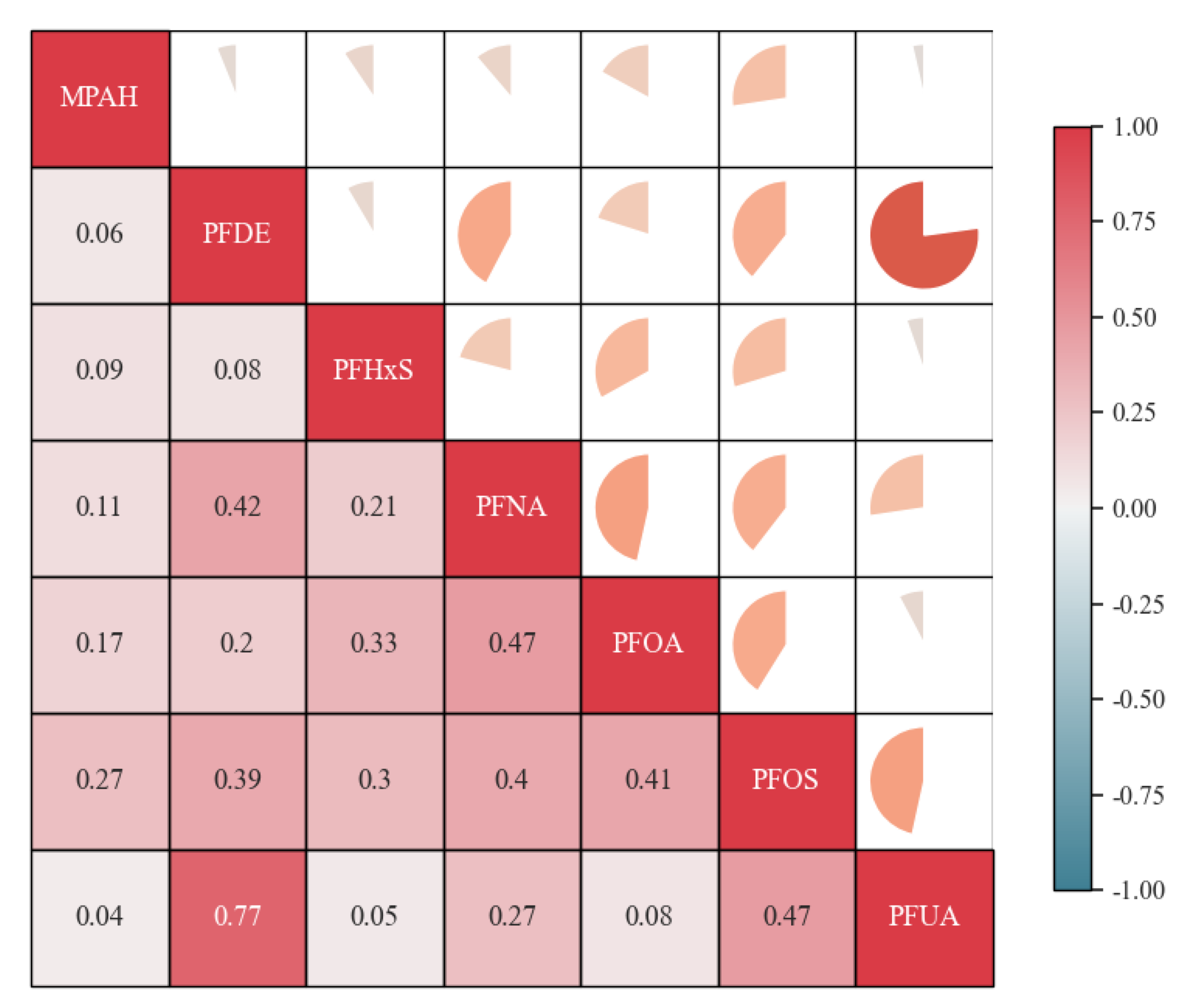

Supplement: Supplementary Figure S2 — Correlation heatmap of serum PFAS concentrations. [file Image_2.tif]
